# Strongly Divergent Impact of Adherence Patterns on Efficacy of Colorectal Cancer Screening: The Need to Refine Adherence Statistics

**DOI:** 10.14309/ctg.0000000000000399

**Published:** 2021-09-10

**Authors:** Thomas Heisser, Rafael Cardoso, Feng Guo, Tobias Moellers, Michael Hoffmeister, Hermann Brenner

**Affiliations:** 1 Division of Clinical Epidemiology and Aging Research, German Cancer Research Center (DKFZ), Heidelberg, Germany;; 2 Medical Faculty Heidelberg, University of Heidelberg, Heidelberg, Germany;; 3 Division of Preventive Oncology, German Cancer Research Center (DKFZ) and National Center for Tumor Diseases (NCT), Heidelberg, Germany;; 4 German Cancer Consortium (DKTK), German Cancer Research Center (DKFZ), Heidelberg, Germany.

## Abstract

The performance of colorectal cancer (CRC) screening programs depends on the adherence to screening offers. However, identical adherence levels may result from varying patterns of the population's screening behavior. We quantified the effects of different adherence patterns on the long-term performance of CRC screening for annual fecal immunochemical testing and screening colonoscopy at 10-year intervals.

**METHODS:**

Using a multistate Markov model, we simulated scenarios where, while at the same overall adherence level, a certain proportion of the population adheres to all screening offers (selective adherence) or the entire population uses the screening offers at some point(s) of time, albeit not in the recommended frequency (sporadic adherence). Key outcomes for comparison were the numbers of prevented CRC cases and prevented CRC deaths after 50 simulated years.

**RESULTS:**

For screening with annual fecal immunochemical testing at adherence levels of 10%–50%, ratios of prevented CRC cases (CRC deaths) resulting from a sporadic vs a selective pattern ranged from 1.8 to 4.4 (1.9–5.3) for men and from 1.7 to 3.6 (1.8–4.4) for women, i.e., up to 4–5 times more CRC cases and deaths were prevented when the population followed a sporadic instead of a selective adherence pattern. Comparisons of simulated scenarios for screening colonoscopy revealed similar patterns.

**DISCUSSION:**

Over a lifelong time frame, large numbers of irregular screening attendees go along with much larger preventive effects than small numbers of perfectly adhering individuals. In clinical practice, efforts to reach as many people as possible at least sporadically should be prioritized over efforts to maximize adherence to repeat screening offers.

## INTRODUCTION

It is widely accepted that screening for colorectal cancer (CRC) is a very effective and cost-effective approach to reduce CRC incidence and mortality (1–4). Screening strategies most commonly recommended by expert panels and offered in screening programs around the world include fecal immunochemical testing (FIT) every 1 or 2 years and 10-yearly screening colonoscopy, starting age 45 or 50 years and continuing at least up to age 75 years (5). Although these strategies may be effective, the performance of a population-based screening program also depends, to a large extent, on the adherence to screening offers.

Adherence to screening is commonly quantified by the proportion of the population in the eligible age range that is up to date with screening, i.e., has had a FIT within the past 1 or 2 years or a colonoscopy within the past 10 years (6–8). These metrics, however, do not differentiate between situations in which a certain proportion of the population adheres to all screening offers and situations in which a large proportion of the population uses the screening offers at some point(s) of time, albeit not in the recommended frequency. For instance, an adherence pattern where half of the eligible population makes use of an annual FIT screening offer will yield the same overall uptake level as a pattern where the entire population makes use of the offer only every second year. Such hidden variations in utilization patterns could, however, result in considerable differences in the effectiveness of comparably designed screening programs because the incremental benefit of screening is likely higher for first time than for repeated uptake (9).

Although several studies have addressed coverage and participation of CRC screening offers (10,11), the evidence of long-term effects of different adherence patterns is limited (12). We, therefore, performed a simulation study to quantify the expected effects of FIT-based and colonoscopy-based screening offers for 2 distinct patterns of adherence that would go along with seemingly equivalent adherence metrics.

## METHODS

### Multistate Markov Model

For this study, we used the previously developed and externally validated Markov-based Colorectal Cancer Multistate Simulation Model (COSIMO) to simulate the effects of screening for CRC in a hypothetical German population (13). Documentation on the model's structure and data sources used for its development are provided in Supplementary Appendix 1, http://links.lww.com/CTG/A678, including overviews on all model parameters (Supplementary Tables 1–3, http://links.lww.com/CTG/A678). The model's source code is available for download from our website (14).

**Figure 1.**
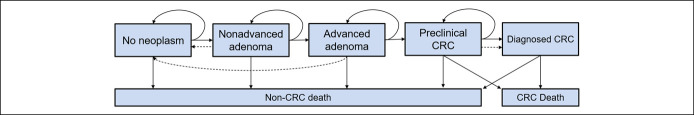
Schematic illustration of COSIMO, the multistate Markov model. Solid lines represent the progression of colorectal disease through the adenoma-carcinoma sequence in the absence of screening. Dashed lines show the movement between states because of the detection and removal of adenomas and the detection of asymptomatic CRC at screening. CRC, colorectal cancer.

Briefly, COSIMO simulates the natural history of CRC based on the process of precursor lesions developing into preclinical and then clinical cancer in a hypothetical population for a predefined number of years. Screening can interfere with the natural history of CRC (Figure 1). The model's natural history assumptions were derived from data of the German screening colonoscopy registry, the world's largest registry of its kind (15–18). CRC-specific mortality rates by modes of detection were estimated using data from a German case-control study with long-term follow-up of patients with CRC combined with German registry data on the proportion of screening-detected cases among all CRC cases (9,18). General mortality rates and average life expectancy were extracted from German population life tables (19).

### Simulations

#### Modeled scenarios

In each simulated scenario, the model population consisted of previously unscreened 100,000 men and 100,000 women aged 50 years at model start. Models were run for 50 years, i.e., up to age 100 years, which allows us to assess effects over a lifelong time frame. First, we performed simulations assuming perfectly adhering populations for maximal offers of FIT and colonoscopy-screening strategies, i.e.,

1. Annual FIT screening from ages 50 to 75 years.
2. Screening colonoscopy at ages 50, 60, and 70 years.

Second, we simulated scenarios with imperfect adherence patterns for both strategies. For the purpose of this study, we defined adherence as

$$\frac{Number\;of\;people\;screened}{Number\;of\;eligible\;people\;invited}\;\times \;\frac{Number\;of\;screening\;tests\;used\;}{Number\;of\;screening\;tests\;offered\;}\;$$

during a given time frame. This definition expands the definition of uptake (participation) by the European Guidelines for Quality Assurance in CRC Screening (20) (the number of people screened divided by the number of eligible people invited) by a second term quantifying the frequency or intensity of (repeated) screening (the number of screening tests used divided by the number of screening tests offered). The extension allows us to assess global adherence levels over an extended period for situations where screening tests are offered in regular intervals, as typically the case in organized screening programs (5).

We defined 2 types of distinct adherence patterns, namely, selective and sporadic adherence. Selective adherence reflects a pattern where a selective proportion of eligible subjects adheres to screening in the recommended frequency. Hence, the second part of the equation will be 1, and the overall adherence for such a pattern equals

$$\frac{Number\;of\;people\;screened}{Number\;of\;eligible\;people\;invited\;}\;\times \;100\%.$$

Sporadic adherence, on the other hand, reflects a pattern where all eligible subjects attend screening irregularly, i.e., not in the recommended frequency. Thus, for a sporadic adherence pattern, the first part of the equation will be 1, and the overall adherence equals

$$100\%\;\times \;\frac{Number\;of\;screening\;tests\;used\;}{Number\;of\;screening\;tests\;offered\;}.$$

More plainly, in the case of selective adherence, the population consists of 2 groups: one group that never uses screening and the other fully adherent group. In the case of sporadic adherence, the entire population uses screening offers but not at the recommended frequency. Although none of these distinct patterns is expected to occur in their pure form in practice, the herein used clear-cut distinction allows us to directly quantify the effects of different adherence patterns for a screening program's long-term efficacy.

We therefore, defined, for both patterns of adherence, sets of scenarios yielding the same overall levels of adherence for the modeled time frame, i.e., 50 years. For instance, for annual FIT screening, the pair of 50% annually adherent subjects (i.e., selective adherence) vs 100% of subjects adhering every 2 years (i.e., sporadic adherence) will correspond to a level of 50% adherence, the pair of 33% annually vs 100% every 3 years will correspond to a level of 33% adherence, and so forth. Similarly, for an offer of screening colonoscopy every 10 years, the pair of 67% fully adherent subjects to screening at ages 50, 60, and 70 years vs 100% adherent subjects to screening at ages 50 and 60 years (or 50 and 70, or 60 and 70) will correspond to a level of 67% adherence, and the pair of 33% at ages 50, 60, and 70 years vs 100% at age 50 years (or 60, or 70) will correspond to a level of 33% adherence.

#### Outcomes

For each scenario, we assessed the cumulative number of prevented CRC cases and CRC deaths after 50 years and the associated percentage reduction when compared with a scenario without screening. We also calculated the cumulative number of prevented years of potential life lost (YPLL) due to CRC deaths. YPLL is a weighted metric taking the average remaining life expectancy at premature death into account, i.e., deaths at a younger age will be given greater weight than deaths at older age. Finally, to allow a more direct assessment of differences, we determined the ratios of prevented CRC deaths and YPLL given sporadic adherence vs the same outcome parameters given selective adherence.

#### Sensitivity analyses

To explore the impact of uncertainty related to modeled key parameters, all point estimates of the starting prevalences and transition rates were replaced by either the lower or upper limits of the 95% confidence intervals. In addition, we modeled scenarios with screening starting age 45 years to assess the potential impact of earlier screening start.

## RESULTS

**Figure 2.**
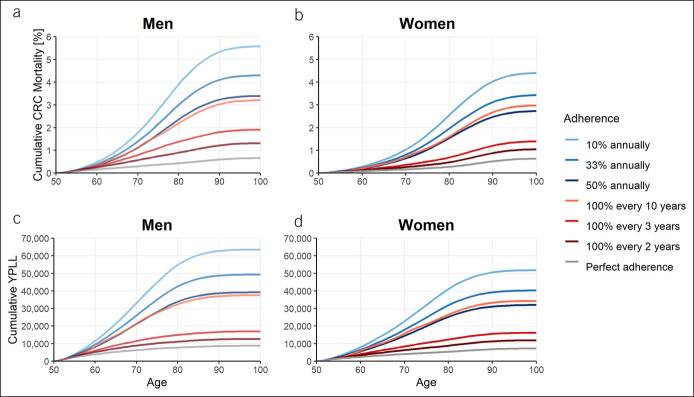
Cumulative mortality and cumulative YPLL due to CRC deaths for annual FIT screening given varying patterns of adherence at defined adherence levels stratified by sex. Cumulative mortality (**a**, **b**) and cumulative YPLL due to CRC deaths (**c**, **d**) for annual FIT screening given varying patterns of adherence at defined adherence levels (10%, 33%, and 50%), stratified by sex. Blue lines: selective adherence (only a part of the population uses screening offers, but in the recommended frequency). Red lines: sporadic adherence (the entire population uses screening offers, but not in the recommended frequency). CRC, colorectal cancer; FIT, fecal immunochemical test; YPLL, years of potential life lost.

**Figure 3.**
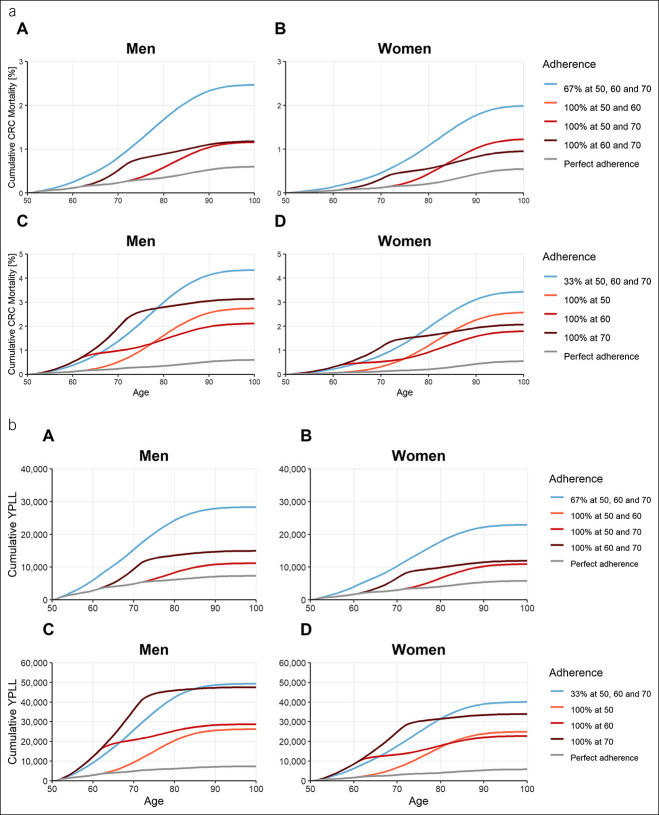
(**a**) Cumulative mortality for screening colonoscopy at ages 50, 60, and 70 years given varying patterns of adherence at defined adherence levels (67%, **a** and **b**; 33%, **c** and **d**), stratified by sex. (**b**) Cumulative YPLL for screening colonoscopy at ages 50, 60, and 70 years given varying patterns of adherence at defined adherence levels (67%, **a** and **b**; 33%, **c** and **d**), stratified by sex. Blue lines: selective adherence (only a part of the population uses screening offers, but in the recommended frequency). Red lines: sporadic adherence (the entire population uses screening offers, but not in the recommended frequency). CRC, colorectal cancer; YPLL, years of potential life lost.

Tables 1 and 2 show differences in simulated outcomes after 50 years, given varying patterns of adherence for screening with FIT and colonoscopy, respectively. Trajectories of cumulative mortality and cumulative YPLL at selected levels of adherence are shown in Figure 2 for annual FIT screening and in Figure 3 for screening colonoscopy at ages 50, 60, and 70 years.

**Table 1.** Differences in long-term outcomes for screening with annual FIT from ages 50 to 75 years, given varying patterns of adherence yielding identical adherence levels

Selective adherence				Sporadic adherence				Ratio sporadic/selective		
Scheme	Incidence reduction	Mortality reduction	YPLL reduction	Scheme	Incidence reduction	Mortality reduction	YPLL reduction	Incidence reduction	Mortality reduction	YPLL reduction
Men										
100% annually	81%	89%	87%	100% annually	81%	89%	88%	1.0	1.0	1.0
50% annually	41%	45%	44%	100% every 2 year	73%	84%	82%	1.8	1.9	1.9
33% annually	27%	30%	29%	100% every 3 year	65%	79%	76%	2.4	2.6	2.6
25% annually	20%	22%	22%	100% every 4 year	58%	73%	70%	2.9	3.3	3.2
20% annually	16%	18%	17%	100% every 5 year	53%	69%	65%	3.3	3.9	3.7
17% annually	14%	15%	15%	100% every 6 year	48%	63%	60%	3.5	4.2	4.1
14% annually	12%	13%	12%	100% every 7 year	43%	57%	55%	3.7	4.5	4.4
13% annually	10%	11%	11%	100% every 8 year	40%	56%	53%	4.0	5.0	4.8
11% annually	9%	10%	10%	100% every 9 year	37%	48%	47%	4.1	4.8	4.9
10% annually	8%	9%	9%	100% every 10 year	36%	48%	46%	4.4	5.3	5.3
Women										
100% annually	79%	87%	87%	100% annually	79%	87%	87%	1.0	1.0	1.0
50% annually	40%	43%	44%	100% every 2 year	67%	78%	79%	1.7	1.8	1.8
33% annually	26%	29%	29%	100% every 3 year	58%	71%	72%	2.2	2.5	2.5
25% annually	20%	22%	22%	100% every 4 year	50%	64%	65%	2.5	3.0	3.0
20% annually	16%	17%	17%	100% every 5 year	46%	61%	60%	2.9	3.5	3.4
17% annually	13%	14%	15%	100% every 6 year	40%	54%	54%	3.0	3.8	3.7
14% annually	11%	12%	12%	100% every 7 year	35%	47%	48%	3.1	3.8	3.9
13% annually	10%	11%	11%	100% every 8 year	33%	47%	47%	3.4	4.4	4.3
11% annually	9%	10%	10%	100% every 9 year	28%	38%	40%	3.2	3.9	4.1
10% annually	8%	9%	9%	100% every 10 year	28%	38%	40%	3.6	4.4	4.5

CRC, colorectal cancer; FIT, fecal immunochemical test; YPLL, years of potential life lost.

**Table 2.** Differences in long-term outcomes for screening colonoscopy at ages 50, 60, and 70 years, given varying patterns of adherence yielding identical adherence levels

Selective adherence				Sporadic adherence				Ratio sporadic/selective		
Scheme	Incidence reduction	Mortality reduction	YPLL reduction	Scheme	Incidence reduction	Mortality reduction	YPLL reduction	Incidence reduction	Mortality reduction	YPLL reduction
Men										
100% at ages 50, 60, and 70 years	84%	90%	90%	100% at ages 50, 60, and 70 years	84%	90%	90%	1.0	1.0	1.0
67% at ages 50, 60, and 70 years	56%	60%	59%	100% at ages 50 and 60 years	76%	81%	84%	1.4	1.4	1.4
				100% at ages 50 and 70 years	73%	81%	79%	1.3	1.4	1.3
				100% at ages 60 and 70 years	69%	78%	66%	1.2	1.3	1.1
33% at ages 50, 60, and 70 years	27%	29%	29%	100% at age 50 years	51%	55%	62%	1.9	1.9	2.1
				100% at age 60 years	58%	66%	59%	2.1	2.2	2.0
				100% at age 70 years	37%	49%	32%	1.4	1.7	1.1
Women										
100% at ages 50, 60, and 70 years	84%	89%	90%	100% at ages 50, 60, and 70 years	84%	89%	90%	1.0	1.0	1.0
67% at ages 50, 60, and 70 years	55%	59%	60%	100% at ages 50 and 60 years	70%	75%	81%	1.3	1.3	1.4
				100% at ages 50 and 70 years	73%	80%	79%	1.3	1.4	1.3
				100% at ages 60 and 70 years	74%	80%	71%	1.3	1.4	1.2
33% at ages 50, 60, and 70 years	27%	29%	29%	100% at age 50 years	43%	47%	56%	1.6	1.6	1.9
				100% at age 60 years	57%	63%	60%	2.1	2.2	2.0
				100% at age 70 years	46%	57%	40%	1.7	2.0	1.4

CRC, colorectal cancer; YPLL, years of potential life lost.

Assuming a perfectly adhering population, both annual FIT screening and screening colonoscopy at 10-year intervals resulted in pronounced reductions of CRC cases (79%–84%), CRC deaths (87%–90%), and YPLL due to CRC (87%–90%) as compared with no screening in both sexes.

### Annual FIT screening

Different patterns of adherence, while at the same overall adherence levels, were associated with considerable differences in screening efficacy. For annual FIT screening at simulated adherence levels of up to 50%, ratios of prevented CRC cases assuming sporadic vs selective patterns ranged from 1.8 to 4.4 for men and from 1.7 to 3.6 for women, i.e., at the same overall level of adherence, in both sexes, up to 4 times more CRC cases were prevented when the simulated population followed a sporadic rather than a selective adherence pattern. Differences were similar for CRC deaths and YPLL (ratios sporadic vs selective, 1.9–5.3 in men and 1.8–4.5 in women). Overall, ratios calculated for outcomes assuming sporadic vs selective adherence tended to be less pronounced for comparably high (e.g., 50%) and more pronounced for low (e.g., 10%) levels of adherence and tended to be higher in men as compared with women.

### Screening colonoscopy at 10-year intervals

Assuming adherence levels of 33% and 67% for screening colonoscopy at 10-year intervals revealed a similar trend as observed for annual FIT screening, i.e., the sporadic scheme yielded overall higher levels of efficacy than the selective scheme. Ratios of prevented CRC cases, deaths, and YPLL for sporadic vs selective adherence were similar across men and women, ranging from 1.1 to 2.2 in men and 1.2 to 2.2 in women. They tended to be higher for men in case of sporadic adherence at younger ages and higher for women in case of sporadic adherence at older ages.

### Sensitivity analyses

Overall, sensitivity analyses using upper and lower limits of 95% confidence interval of starting prevalences and annual transition rates as well as analysis with starting age 45 years yielded comparable CRC risk reductions as well as similar ratios for sporadic vs selective patterns as compared with the base case scenario (Supplementary Tables 4–9, http://links.lww.com/CTG/A678).

## DISCUSSION

This simulation study provided estimates on the efficacy of CRC screening strategies based on varying patterns of the eligible population's screening behavior. Assuming distinct adherence patterns, defined as selective adherence (where only a part of the population uses screening offers but in the recommended frequency) and sporadic adherence (where the entire population uses screening offers but not in the recommended frequency), we found that different patterns of adherence, while at the same overall adherence level, were associated with considerable differences in long-term efficacy. Our findings suggest that substantially more CRC cases, CRC deaths, and YPLL could be prevented when the population followed a sporadic instead of a selective adherence pattern. For annual FIT screening (at uptake levels of 10%–50%) and for screening colonoscopy at 10-year intervals (at uptake levels of 33% and 67%), adopting a population-wide sporadic adherence pattern was estimated to be up to 4–5 times and up to 2 times more effective than a selective pattern, respectively.

### Findings in context

Population-based screening for CRC involves a multitude of complex programmatic issues, reflected in differences in the design of screening programs around the world. Programs vary about the eligible population, targeted age groups, implementation approach, objectives as well as offered screening tests, and intervals (5). Eventually, however, the effectiveness of any program will be driven by 2 main components: the efficacy of the offered screening tests when they are actually used and their uptake by the targeted population.

It remains unclear which screening strategy is the most effective to offer. No direct comparisons of the performance of alternative strategies have been completed, and long-term outcomes of head-to-head studies underway are not expected before the late 2020s (21). Evidence from modeling studies based on perfect adherence, an assumption which reflects the point of view of an individual subject, suggests that screening with colonoscopy at 10-year intervals may be the most efficacious strategy, but other strategies, including annual FIT, do not fall much behind for the reduction of the risk of dying from CRC (22).

From a public health perspective, achieving high levels of population adherence is an additional and particularly important component to be considered. Health authorities may decide to introduce one screening strategy or another, e.g., annual FIT or 10-yearly colonoscopy, as assumed in our study, but the eligible population's uptake can at best be influenced indirectly. The necessary understanding of the mechanisms of adherence to CRC screening offers is constantly evolving. Broadly, the characteristics of eligible population and program design are known to affect the population uptake. For instance, higher participation rates have been reported among women vs men and in those aged older than 60 years vs younger age groups (7). An association was also reported for lifestyle factors and cultural background (10).

A recent study found that countries with nationwide coverage of organized screening programs offering only fecal testing or fecal testing and colonoscopy as alternatives had the highest levels of population adherence (7). Higher uptake of FIT vs colonoscopy was also seen in the first round of the randomized COLONPREV study, comparing 1-time colonoscopy vs biennial FIT (23). Uptake in subsequent screening rounds tends to be high among those adherent to a first invitation (24). Personal invitations with directly mailed FITs lead to higher utilization rates (25). Patient navigation and education were also found to have a significant effect (26).

Differential patterns of screening behavior through several screening rounds have been described previously (27,28). These include consistent screening attendees and consistent nonresponders (reflected in the selective adherence pattern in our study), as well as intermittent attendees with late entry, drop out, or intermittent participation (reflected in the sporadic adherence pattern). Estimates on the distribution of these behavioral patterns within a specific population are naturally difficult, given the limited evidence and large heterogeneity of population characteristics. In the few available studies, approximately 40%–50% of studied subjects were consistent screeners and 20%–30% consistent nonresponders, with the remainder following intermittent participation patterns (24,27–30).

Although it has been acknowledged that differences in adherence patterns need to be taken into account when interpreting the trial results (22), the evidence on the actual impact on long-term outcomes is scant. A retrospective cohort study found that not being up to date in screening increased the risk for CRC death nearly 3-fold (12). A previous modeling study suggested that approximately 60% of US CRC deaths are attributable to the nonuse of screening (31). To our knowledge, no previous study has assessed differential longitudinal adherence patterns for their impact on long-term outcomes. Our study adds to the literature that, from a societal perspective, large proportions of the population making sporadic use of screening offers will be substantially more beneficial to achieve sustained reductions of CRC mortality and YPLL due to CRC deaths than small proportions of the population using screening offers at the recommended frequency.

Finally, it should be noted that the simulated scenarios purposely reflect extreme scenarios where populations would only adhere in either a selective or a sporadic fashion. First, as previously mentioned, evidence to inform the model on real-world longitudinal adherence patterns over a lifelong time frame is limited. Second, such extreme scenarios allow us to assess principle, direction, and range of potential differences between both types of adherence patterns. Although the simulated patterns are unlikely to be found in practice in pure form, we believe the study of extremes to be the best approach for illustrating the effects of varying patterns of longitudinal adherence.

### Implications for CRC screening

Clearly, optimal protection from CRC can only be achieved by perfect adherence. However, even in countries with well-organized screening programs, such as the Netherlands, where up to 60% of the eligible population regularly attend screening (32,33), full adherence remains far out of reach. Therefore, our findings should encourage health authorities to concentrate efforts on promoting a broader reach of screening in the eligible population, paying particular attention to better target and reach notoriously more difficult-to-reach population groups, such as less educated or otherwise socially disadvantaged groups (34).

Translated to clinical care, this implies that efforts to reach those previously unscreened should be prioritized over efforts to maximize uptake of repeated screening measures. Raising patient awareness on the benefits even of sporadic screening will be pivotal, e.g., by information campaigns, as well as identifying and thoughtfully communicating with those consistently nonresponding. Where feasible, personal invitations, ideally combined with directly mailed FITs, should be used. Offering patient navigation or educational measures may also be supportive for achieving higher uptake. By illustrating the strong impact on individual risk reductions even by only sporadic use of screening, our findings will also support the communication with screening-hesitant patients.

Finally, our study points to the need for a broader reflection on the long-term effects of adherence mechanisms. In the United States, the National CRC Roundtable had agreed on a goal of 80% screened (35). The European guidelines consider a minimum uptake of 45% as acceptable but recommend aiming for at least 65% (20). Suchlike aspirational targets typically refer to the number of people screened as a proportion of all people who are invited to attend a specific screening offer within population-based screening programs, e.g., that has had a FIT within the past 1 or 2 years, or a colonoscopy within the past 10 years (6–8). Our results suggest expanding such adherence metrics by additional indicators taking adherence patterns over multiple rounds of screening into account whenever the data allow to do so. Ideally, such metrics should inform on effective adherence, e.g., by reporting the proportion of subjects who ever used a screening test, or the rescreening adherence when compared with 1 or several previous rounds of screening.

### Strengths and limitations

Specific strengths and limitations of COSIMO have been described previously (9,13). Briefly, a major strength of our model is the use of input parameters derived specifically from the German general population using the world's largest screening colonoscopy registry. Furthermore, the model was subjected to a thorough assessment of its external validity and was found to adequately predict colorectal neoplasm prevalences and incidences in a German population, with estimated patterns of the effect of screening colonoscopy resembling those seen in the registry data and real-world studies (13). Major limitations concern model simplifying assumptions and uncertainties related to input parameters where evidence was limited, for instance regarding transition rates for age groups 50–54 years and 80+ years, true screening test performance characteristics in Germany, and potential differences between sexes in this respect.

In summary, long-term preventive effects of CRC screening programs may vary substantially because of patterns of the population's screening behavior over time. In an average-risk screening population, large numbers of irregular screening attendees go along with much larger preventive effects than small numbers of consistently perfectly adhering individuals. In clinical practice, efforts to reach as many people as possible at least sporadically should be prioritized over efforts to maximize adherence to repeat screening offers. Adherence statistics should be refined to better reflect effective adherence.

## CONFLICT OF INTEREST

**Guarantor of the article:** Thomas Heisser, Msc

**Specific author contributions:** H.B. and T.H.: designed the study and developed the methodology. T.H.: conducted the statistical analyses and drafted the article. All authors critically reviewed the article, contributed to its revision, and approved the final version submitted. The researchers are independent from funders. All authors had full access to all of the data (including statistical reports and tables) used for the study and can take responsibility for the integrity of the data and the accuracy of the data analysis.

**Financial support:** Financial support for this study was provided in part by a grant by the German Federal Ministry of Education and Research (grant number 01GL1712). The funding agreement ensured the authors' independence in designing the study, interpreting the data, writing, and publishing the report. T.H. was supported by the Helmholtz International Graduate School for Cancer Research at the German Cancer Research Centre (DKFZ).

**Potential competing interests:** The authors declare that they have no conflict of interest.

**Study Highlights**

**WHAT IS KNOWN**

✓ The performance of screening programs for colorectal cancer depends on the adherence to screening offers.
✓ The evidence of effects of different longitudinal adherence patterns (e.g., consistent or sporadic uptake) on long-term outcomes is limited.

**WHAT IS NEW HERE**

✓ In a simulated population, at identical overall participation levels, large proportions of the population making sporadic use of screening achieved up to 4–5 times higher incidence and mortality reductions than small proportions of the population using screening offers at the recommended frequency.

## Supplementary Material

SUPPLEMENTARY MATERIAL
